# Methods and strategies to promote academic literacies in health professions: a scoping review

**DOI:** 10.1186/s12909-022-03288-9

**Published:** 2022-06-01

**Authors:** A Klarare, I-B Rydeman, Å Kneck, E Bos Sparén, E Winnberg, B Bisholt

**Affiliations:** 1 Department of Women’s and Children’s Health, Healthcare Sciences and e-Health, Uppsala University, Uppsala, Sweden; 2 Department of Health Care Sciences, Marie Cederschiöld University, Stockholm, Sweden; 3 Department of Healthcare Sciences, The Swedish Red Cross University College, Huddinge, Sweden

**Keywords:** Higher education, Academic literacies, Undergraduate health education, University students, Scoping review

## Abstract

**Background:**

Universities enroll students from diverse backgrounds every year, with 300 million students expected in higher education by 2025. However, with widening participation, increasing numbers of students enrolling in higher health education and future health professions will be underprepared to meet demands of academic literacies, i.e. ability to read, interpret and critically evaluate academic texts and communicating the understanding verbally or in writing. The aim of this scoping review was to describe and explore methods and strategies to promote development of academic literacies.

**Results:**

Thirty-one relevant studies were included and analyzed according to scoping review guidelines. The results showed four strategies: (1) *integrating learning activities to develop academic literacies in the regular curriculum*, (2) *changing the course design with new methods for teaching and learning*, (3) *establish collaborations amongst academics and librarian faculty*, and (4) *adding courses or foundation year focusing on development of academic literacies*. The results are discussed in light of the United Nations Agenda 2030 Sustainable Development, Goal 4, Quality Education, and widening participation.

**Conclusions:**

Aspects of widening participation and inclusion in higher education have been debated, and increasing numbers of students from diverse backgrounds are expected to enter health studies in higher education. We encourage integration of teaching and learning activities targeting parallel learning of course materials and development of academic literacies, beyond study skills. Embracing epistemic complexity and diversity as well as choosing strategic work with academic literacies may provide a starting point toward realizing sustainable development goals and widening participation.

**Supplementary information:**

The online version contains supplementary material available at 10.1186/s12909-022-03288-9.

## Introduction

Numerous students enter higher education with relatively little or no experience of academic studies [1], and previous education does not ensure that students are sufficiently equipped to meet requirements of academic literacies [2]. Moreover, formal requirements may differ between higher education institutions. Higher education entails adapting to new ways of understanding, interpreting and organizing knowledge [3], in parallel with effectively developing academic competence; therefore, assuming that students have the required academic literacy skills for higher education is unwise [4]. Academic literacies are crucial for students as the ability to read, interpret and critically evaluate texts forms the foundation of understanding academic texts, and in turn communicating their understanding either in written or spoken form [2]. The United Nations 2030 Agenda for Sustainable Development was adopted by all member states in 2015 with the explicit aim to strategically work for peace and prosperity for people and the planet [5]. The agenda is stated in terms of 17 goals comprising a global call to action. Sustainable development goal (SDG) 4 highlights inclusive and equitable quality education and the promotion of life-long learning for all.

The term academic literacy or literacies can be expanded and shaped by the context of the particular discipline in which it is used [6], highlighting that mastering academic writing in different genres, developing skills in critical thinking and appraisal is fundamental to all academic disciplines [7]. There are several definitions of academic literacies, and Lum et al. [8] emphasize that in health professionals programs, academic literacies in a broader perspective are not strictly cognitive activities, but also include interpretation and producing a variety of academic texts within important social contexts of their professional discipline. Included in definitions of academic literacies are the ability to write for different purposes, target groups and occasions, as well as accessing, interpreting and evaluating information, utilizing critical thinking, which is reflected in performance and creation of new texts and knowledge [9]. Critical thinking ability is strongly associated with academic writing [7].

Lea and Street highlight three models of academic literacies [3, 10], namely study skills, academic socialization and academic literacies. These models are not mutually exclusive, nor do they replace each other, rather, they broaden the scope, build on each other and can facilitate a more comprehensive understanding of students’ writing and literacies in higher education. The study skills model focuses on the ‘doing’ associated with writing, often in terms of grammar and spelling, the formal features of language. Behavioristic principles have inspired the model and learning is about transfer of knowledge. The second model, academic socialization, builds on study skills and also considers student acculturation into a new context. The subject-based discourse and genres of writing are considered as students learn new ways of talking, writing and thinking within the specific context [3]. The process is a part of the socialization into academia, and learning different ways for knowledge construction in a new context [3, 10]. This model aligns with constructivism and situated learning theory. The critique for these two models is that they may be simplistic and lead to more shallow understanding. The third model, academic literacies, takes study skills and academic socialization into consideration, however, also adding meaning making, power dynamics and identity, to the widen the scope. This model is influenced by social and critical linguistics and sociocultural learning theory, involving epistemology and social processes among people, institutions and social identities [3]. Both students and teachers learn from each other through meaning making and reflecting on identity in literary practices. Lea and Street [10] further emphasize that higher education institutions are sites of discourse and power.

Every year universities and higher education institutions enroll new students from diverse backgrounds [11], with 300 million students expected world wide in the year 2025 [12]. Within health professions, applications to study medicine and nursing have increased during the COVID-19 pandemic. Most likely, increasing numbers of health students with aspirations for higher education will continue to enroll, and subsequently, they will meet expectations of using academic literacies to successfully complete their studies. However, being able to beforehand prepare for all expectations within higher education studies is difficult [13], and many students come underprepared for the requirements of academic literacies, especially in light of Lea and Street’s [3] academic literacies model encompassing epistemology and identity, rather than mere study skills and academic socialization. Taken together, universities find themselves challenged to develop programs, courses and curricula to meet students’ various needs of support in relation to academic literacies. In health programs, international recruitment initiatives contribute to universities enrolling students with diverse language and linguistic backgrounds, some studying in their second or third language.

An overview of strategies and methods used by higher education institutions in health professions, to promote development of academic literacies in enrolled students warrants exploration and systematic compilation. There are multiple studies of screening and retaining students [14–16] or identifying students likely to succeed [17, 18]. However, in line with SDG 4 [5] and widening participation, this scoping review aimed to describe and explore methods and strategies to promote development of academic literacies in higher education health programs, using the following research questions:

1. What methods and strategies are used in higher education to promote development of academic literacies?
2. What is known about the effectiveness of utilized methods and strategies?
3. How do university health students experience methods and strategies to promote development of academic literacies?

## Methods

This was a scoping review in accord with guidelines stated by Arksey and O’Malley [19], and comprised the five steps: identifying the research question, identifying relevant studies, study selection, charting the data, and collating, summarizing and reporting the results. The scoping review format was selected to provide an overview and summarize research findings to potentially guide design of future studies in the area. The reporting of this study was guided by the Preferred Reporting Items for Systematic Reviews and Meta-Analyses extension for Scoping Reviews (PRISMA-Scr) checklist [20].

### Identifying relevant studies

In June 2020, a systematic search was performed in five databases: *Education Source*, and *Education Resources Information Center* (ERIC) [education], *Public/Publisher MEDLINE* (PubMed) and *Cumulative Index to Nursing and Allied Health* (CINAHL) [medical/healthcare], and *Web of Science* (cross-disciplinary). The search strategy was broad to capture a variety of studies focusing on aspects of academic literacies, up to June 21, 2020. The search strategy is described in Table 1. Reports were limited to the English language.

**Table 1:** Search strategy

Group	Search terms
1	“higher education” OR universit* OR college* OR undergraduate OR Bachelor* OR Baccalaureate* [subject, title, abstract]
2	“nursing students” OR “nursing education” OR “health science students” OR “Health occupations students” [subject, title, abstract]
3	“Academic achievement” OR “Academic ability” OR Aptitude OR “academic performance” OR Educability OR “Developmental Studies Programs” OR “College student development programs” OR “remedial programs” OR “Services for college students” OR “Services for students” OR “student support” OR “academic support” OR “learning support” OR “Transitional Programs” OR “support service” OR “support strategies” OR “Study skills” OR “Study skill” OR “academic writing” OR “Writing centers” OR “writing programs” OR “writing laboratories” OR “writing workshops” OR “Reading (Higher education)” OR “reading improvement” OR “reading centers” OR “reading strategies” OR literacy [subject, title, abstract]
	1, 2 and 3 combined in searches

Inclusion criteria were empirical, peer reviewed studies regarding methods, strategies and programs to promote academic literacies in university settings. Systematic reviews, editorials, commentaries and non-empirical studies were excluded, as were pre-university programs and grey material, to include only academic primary studies, see Table 2.

**Table 2:** Inclusion and exclusion criteria

Criterion	Inclusion	Exclusion
*Types of studies*	Qualitative, quantitative, and mixed methods empirical studies in peer-reviewed journals	Letters, comments, conference abstracts, editorials, doctoral theses, or any type of reviews
*Time period*	All publications to June 21, 2020	Published after June 21, 2020
*Language*	English	Other languages
*Participants*	Undergraduate students in higher education, such as university or university college	Students in high school or upper secondary school; pre-university programs; graduate students
*Phenomenon of interest*	Academic literacies; pedagogies, methods or strategies to develop or promote academic literacies	Computer skills training; pre-university classes; information literacy programs
*Types of outcomes*	Influences on academic literacies; course results, grades, student satisfaction, lecturer reflections	No outcomes reported

The reference lists of included studies were hand searched to identify additional studies for the review.

### Study selection

**Fig. 1:**
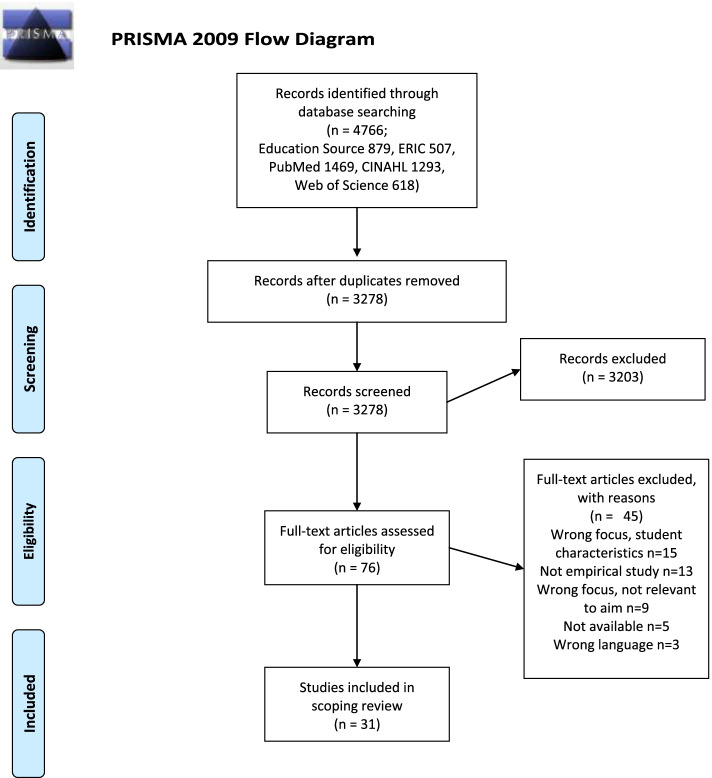
PRISMA 2009 flow diagram

Pairs of authors independently screened titles, abstracts and articles for study inclusion using Rayyan, a mobile, web-based application for systematic reviews [21]. The first step comprised screening titles and abstracts (*n* = 3278) to identify relevant studies for full-text screening, each result was independently screened. Next step comprised comparison and discussion to reach consensus among pairs of authors and choosing studies for full-text screening and assessment. A standardized template was used for full-text screening comprising the research questions, and space to describe study characteristics, i.e. aim, design, sample characteristics, data collection, data analysis, ethical considerations and intervention, see Additional file 1. During full-text screening (*n* = 75 studies), studies that did not meet the study aim were excluded. If pairs of authors disagreed, a third author acted as arbitrator to reach consensus. No studies were excluded due to lack of quality, however, lacking explicit ethical consideration or tranparency in method descriptions was noted. Finally, 31 studies were included in the review. See summary of the selection process in Fig. 1, PRISMA protocol.

### Charting the data

During the process of charting the data, pairs of authors extracted data from the studies using a standardized template. The template was created with space for findings relative to the research questions, other relevant findings, key message and comments, see Additional file 1.

### Collating, summarizing and reporting

Data from the included studies were organized into a matrix for an overview of results. The extracted results were read multiple times to identify patterns, similarities and differences in interventions, methods and strategies to promote academic literacies in higher education [19]. An inductive process was adopted to group emerging findings thematically in categories [22]. First, second and last authors discussed findings and agreed on categories before they were presented and discussed with the other authors. This iterative process, requiring researcher reflexivity, was crucial for the broad approach and aiming to present comprehensive results regarding the range of research relevant to promoting academic literacies in higher education institutions.

## Results

A total of 31 studies were included in the scoping review (Fig. 1; Additional file 2). No studies were identified or included through hand searching. The studies were conducted in Australia (*n* = 5), Bahrein (*n* = 1), Canada (*n* = 1), China (*n* = 1), Iran (*n* = 1), Malaysia (*n* = 1), New Zealand (*n* = 2), Pakistan (*n* = 1), South Africa (*n* = 1), Taiwan (*n* = 2), the United Kingdom (*n* = 5), and the United States (*n* = 10). The sample size of included studies ranged from five to 812 (median 65, mean 123), with a total of 3823 participants, all university students with the exception of 19 persons, who were teachers, tutors or librarians. The undergraduate students were enrolled in bioscience (*n* = 60), medicine (*n* = 363), nursing (*n* = 2298), occupational therapy (*n* = 199), pre-nursing (*n* = 53), and unspecified health sciences (*n* = 803). Study designs included action research (*n* = 5), case studies (*n* = 6), experimental studies, either with or without a control group (*n* = 14), mixed methods (*n* = 4), qualitative studies (*n* = 1), and quantitative, cross-sectional studies (*n* = 1). See Additional file 2 for an overview of included studies.

To answer the aim and research questions of this scoping review, the results are presented in two thematic groupings: *Overview of methods and strategies to promote academic literacies*, and *Outcomes and student experiences of interventions.*

### Overview of metods and strategies to promote academic literacies

Thirty-one studies (see Additional file 2) reported methods and strategies that higher education institutions used to create opportunities for developing academic literacies. Eleven studies described integrating development of academic literacies in the regular curriculum [23–33], where some of these specifically highlighted changing the course design or pedagogies to embed or integrate academic literacy competencies [23, 24, 27–29, 34]. Studies described changing the program or course design to: team-based learning [23], problem-based learning [34], flipped classroom [35], assignments that require critical thinking [36], literacy enrichment of program [25, 30, 31], integrating language focus in subject content [26, 27], and integrating information literacy in collaboration with librarians as instructors [28, 33, 37]. Daniels and Jooste [24] described adding an additional foundation year to the nursing program, where the first year was extended to two years instead of one, giving students more time to study the material, and to develop study skills and academic literacies.

A common method described in eight studies was using a workshop format, with smaller groups of students, focusing on academic writing and study skills [33, 37–43]. In addition to workshops, two included peer instruction, review and learning [37, 42]. Another option for promoting academic literacies was offering a parallel course or program, for example English language support to students with other first language [44], a writing intensive course to improve science-specific writing skills [45], additional workshops targeting specific issues raised by students [43], and a retention course focusing on study skills and critical thinking competencies [41].

Interventions to promote academic literacies were either concentrated to a specific course [23, 25, 27–30, 34–36, 39, 42, 43, 45, 46], to semesters one (first half of first year) or four (second half of second year) [32], to the nine first months of university for underprivileged students [41], or by extending the first year to two years for underprivileged students [24]. In a few studies placement and inclusion were not specified or unclear [31, 37, 38, 40, 47–51].

Using digital tools and resources to support development of academic literacies was described in six studies: electronic technology for academic writing [47], designing a phrase bank with and for students [48], an interactive web-based academic writing tool [49], using an artificial intelligence tool to address academic writing [50], web-based support such as tutorials, online discussions [32], and a web-based nursing information literacy course [46].

### Outcomes, effectiveness and student experiences of interventions

The format for describing outcomes comprised both descriptive data from surveys or questionnaires as well as from group or individual interviews. Seven studies described outcomes of interventions using statistics, in relation to improved grades or academic progress [23, 25, 34, 35, 38, 41, 43], and one each of increased confidence with APA referencing [28], improved writing self-efficacy [51], improved written communication [27], and improved academic literacy competency [31]. These diverse outcome measures did not allow inferences regarding effectiveness.

Considering students’ perceptions/opinions of various interventions, increased confidence was one of the most commonly reported outcomes, present in eight studies [28, 31, 36, 40, 41, 43, 45, 52]. Students also reported learning and understanding subject matter and course content better from flipped classroom pedagogy [35], from using poetry to better understand complex phenomena [39], and satisfaction with increased writing skills [25], as well as using a phrase bank for writing support [48]. Integrating reading and writing skills into the academic curriculum developed students’ understanding [30] and in an example of web-based learning, students expressed effective communication and knowledge building, which in turn promoted self-learning [46].

Seven studies reported that students especially appreciated interactions and face to face time with academic faculty, lecturers, tutors and librarians [28, 33, 37, 40, 43, 50, 53]. Furthermore, students appreciated academic faculty providing constructive feedback and clarifying expectations within courses. [29, 33]. Students also valued a variety of forms for learning [44], small group learning [53] and interactive aspects resulting in more comprehensive learning [35]. In three studies, students highlighted peer and collaborative learning for successful studies [37, 41, 42].

Fourteen studies described interventions available to all students in the course [23, 25, 28, 29, 31, 32, 34, 37, 38, 42, 45, 46, 50, 52], and other specifically targeted student groups like Chinese nursing students in the United States [53], minority or underrepresented students [47, 50], students with cultural and linguistically diverse backgrounds and language support needs [40, 44], and underpriveleged students [24, 41]. These represent different strategies within higher education for addressing student skills and development of academic literacies.

## Discussion

To promote academic literacies in health education, results indicate four groupings of strategies: [1] integrating learning activities aimed at developing academic literacies in the regular curriculum, [2] changing the course design and pedagogies with new methods to facilitate development of academic literacies, [3] establishing collaborations among academics and librarian faculty, or [4] adding parallel courses or an extra foundation year focusing on the development of study skills and academic literacies. Students especially appreciated small group learning in workshop format, elements of peer learning and digital tools that could be accessed at will. Also mentioned were constructive feedback and clarifying expectations as well as a variety of learning formats. The common measures of outcomes in relation to strategies were [1] increased grades or academic progress, [2] increased confidence, and [3] improved academic literacies, operationalized in terms of referencing, writing skills, learning, understanding, and competence. Regarding effectiveness of the included studies, the character of outcome data did not allow making inferences in this area.

This scoping review summarizes and collates research results focusing on development of academic literacies in health education with regards to methods and strategies, outcomes, and student experiences. Even though aspects of widening participation and inclusion in higher education have been debated for years, and considering the fact that increasing numbers of students from diverse backgrounds are expected [12], there does not seem to be consensus nor clear evidence as to which approaches or strategies higher education institutions should adopt. Many of the evaluated studies in this review are examples of initiatives from individual higher education institutions, where students were offered various alternatives with unclear results, and subsequently, potential for generalizability of findings is unclear. Our findings identified no studies with academic literacies training or practice interwoven throughout the whole curriculum. Furthermore, academic literacy is often equated with academic writing, focusing on the ‘doing’ of writing. However, academic literacies in a wider perspective comprise elements of thinking, doing, being, reading and writing specific to academic context [54]. Also, in light of the academic literacies model by Lea and Street [3, 10], relying on study skills and adding academic context socialization is not enough. The whole institutional and epistemological context must be interconnected for students to navigate higher education and successfully participate in and contribute to the academic community.

### Sustainable development goal 4: Quality Education

One of the targets in SDG 4 is to ensure equal access to affordable and quality education, including university level (target 4.3). At present, the trend with widening and increased participation in higher education keeps rising worldwide [11], and has accelerated from 10% in the 1990’s with a future expected gross tertiary enrollment rate close to 50% in most regions. The widening participation in higher education through equal access has been advocated by governments and operationalized in policies [55]. The general idea is to improve participation of students from various equity groups, such as mature learners, second language learners, persons with lower socioeconomic background, and/or ethnic minorities, both with regards to access and to success. The rationale provided for the widening particpation policies is often described in terms of a higher attainment level for citizens and improving national outcomes, such as health and gainful employment. In the context of nursing education, a literature review indicates that the meaning of widening participation has been unclear and that universities have focused on student diversity, rather than changed educational practice to meet the needs of new student groups [56]. Nonetheless, higher education institutions catering to diversity have been found to make a difference, with regards to stratification of students, and have been found more socially inclusive than more traditional ‘elite’ universities [11].

The link between higher education and sustainability may be suggested as preparing students for the unknown, as in learning generic competencies to meet and to be prepared for the future [57]. With a constantly changing and evolving world, students are expected to live and act in supercomplex realities, and Barnett [53] argues that universities are meant to enable individuals to prosper in fluid or ever-changing situations. However, with widening participation, increasing numbers of students enrolling in higher education will be underprepared to meet demands of academic literacies [4], and subsequently may struggle with completing their academic studies and thus not enabled to prosper.

### Widening participation in higher education

Our findings indicate that higher education health institutions struggle with meeting the needs of students and in line with initiatives of widening participation, higher education institutions are encouraged to prepare for and to welcome diverse students from stratified backgrounds and circumstances [11]. The Lea and Street academic literacies model [3, 10] emphasizes a broader scope for addressing students’ engagement and academic literacies in the academic community by highlighting meaning making, power relationships and identity. For first generation higher education students, students with another first language than the majority language and students from underrepresented populations, this scope potentially presents an even greater challenge [8].

Having a higher education degree is a door opener and allows improving career prospects and the bettering of oneself [58], and parents often hope that their children will continue to higher education [11]. However, academic achievement also correlates with having parents with higher education and parental education level is linked to societal expectations for higher education attendance [59]. Sociodemographic advantage or disadvantage is linked to choices in higher education [60], with social class and level of economic risks found as determining choice [61]. However, there are positive findings of educational resilience in students from lower socio-economic circumstances [60], and further, that previous academic performance, university experience and working status are more significant than socio-economic background. Adding to this, social structures strongly determine to what extent individuals can mobilize their abilities, skills and knowledge for agency, i.e. taking action to improve their situation [62]. Taken the inherent complexities, both for individual prospective students and for higher education institutions as discussed above, the crucial question remains, how can the higher education system advocate for and make opportunities for student growth and development more fair and equitable?

### Adopting a comprehensive, systematic and inclusive approach

The results of this review indicate that the strategies and methods to promote development of academic literacies seem conducted in the spirit of good intentions and trial and error. Furthermore, that outcome measures like increased grades, academic progress or confidence are difficult to compare, and initiatives have generally not been replicated in other settings. Hence, drawing robust conclusions and providing generalizable recommendations of strategies and methods is challenging [63, 64]. In light of SDG 4 and equal access to quality university education, it is imperative that higher education institutions in health sciences adopt a comprehensive, systematic and supportive approach in welcoming stratified and diverse student populations. There is no easy remedy or quick fix and these are incredibly complex issues, however, a systems approach is a crucial start so that it is not up to the individual students to both be able to analyze what they are missing and what they need to do to rectify the situation. Neither should it only be up to individual higher education employees to find solutions, rather a systems level analysis and approach is called for, especially in light of the world wide shortage of qualified health professionals.

Traditions of contemporary higher education institutions are founded on conventional epistemological frames that shape teaching, learning and research [65], often holding a view of academia as a priviledged status for a few, not necessarily for the masses. Higher education institutions today are both locally situated and globally connected, calling attention to the already existing fluidity within the higher education sector; and further highlighting the need for knowledge decolonization. Diversity and epistemic complexity, in other words, acknowledging various forms of knowledge, is suggested by Seats [65] to lead the way. This is supported by the academic literacies model as described by Lea and Street [3, 10]. Moving from theoretical reasoning to higher education practice, Ryder et al. [66] show that higher education faculty who encourage exploration of diversity and different cultures significantly influence students’ openness to diversity. Another aspect is producing higher education graduates prepared to explore diverse perspectives and worldviews [66], potentially of value for a peaceful and prosperous world.

Finally, the emerging discourse of knowledge democracy highlights the link between the relationship of knowledge to a more equitable world [67]. Knowledge democracy is described as an interrelationship of phenomena, highlighting the importance of multiple existing epistemologies of knowledge such as organic and spiritual, or knowledge of marginalized and excluded people. Furthermore, that knowledge is created and represented in multiple forms, and also, that knowledge is a powerful tool for deepening democracy and for creating a more equitable world. This description of knowledge democracy aligns Lea and Street’s model of academic literacies [3], highlighting a variety of writing practices and implicit assumptions of what represents acceptable knowledge. Higher education institutions are sites of discourse and power, presenting an arena for development of study skills, academic socialization and development of academic literacies. Positionality is closely linked to identity and having thoughts or reflections on a topic does not come out of vacuum. If higher education insitutions embrace this shift of epistemic complexity and diversity, choosing systematic work with academic literacies beyond study skills, this would be an excellent first step toward meeting Agenda 2030 goals and promoting peace and prosperity for people and the planet [5]. These are lofty goals in a complex reality and may even seem unattainable, however, doing something, trying and taking the first step is a worthy endeavor.

### Strenghts and limitations

This study utilized a scoping review design [19] to systematically explore methods and strategies within health professions education to promote academic literacies. The research group adhered to existing guidelines for undertaking a scoping review, PRISMA-Scr [20], striving for a transparent approach to enable readers to assess study reliability and trustworthiness. A team-based approach was used in the study for representative expertise [68], with two independent evaluations throughout the review process.The searches were conducted in five databases with different characteristics and subject focus to capture a broad range of studies. The search string included nursing and health science or health occupation, however not specifically naming other health professions, which may have skewed the results. Furthermore, the scope of countries where studies were conducted is limited, with only twelve countries, and therefore it is challenging to make inferences or policy recommendations. In line with recommendations for scoping reviews, quality of included studies was not evaluated. However, it is evident in the range of study designs, rationale and methodology that quality varied and subsequently, that making general inferences is challenging. Finally, including studies only in the English language may have excluded relevant publications in other languages. Arksey and O’Malley [19] suggest an optional consultation exercise at the end of a scoping review and including stakeholders in the process could have contributed additional perspectives during the review process.

### Implications for teaching

The results of the review offer some insights into how universities can assist in creating inclusive learning environments for their increasingly diverse student populations. Academic literacies comprise multiple competencies with a core of critical thinking and communication [8], the three models of study skills, academic socialization and academic literacies [3]; and developing academic literacies is a process over time. Complex higher order cognitive activities, inherent in academic literacies, are not taught nor learned in one instance, rather they require perseverance and practice. Therefore, firstly, we encourage higher education institutions to create ample opportunities for practice through integrating teaching and learning activities targeting simultaneous learning of course materials and development of academic literacies. Secondly, we encourage teamwork and interprofessional collaboration in design and development of these teaching and learning activities. For example, including librarians as well as teaching and learning specialists on the faculty teams, involved from conceptualization of curricula and courses, to giving courses and evaluating outcomes. We hope the recommendations offered are able to assist higher education institutions to ensure that they are better prepared to meet the needs of students in health professional programs while also establishing that students develop and learn the requisite academic literacies that will be beneficial for their studies.

## Conclusions

Aspects of widening participation and inclusion in higher education have been debated for years, and increasing numbers of students from diverse backgrounds are expected to enter higher education institutions. However, there is no consensus regarding which approaches or strategies higher education institutions should adopt to promote development of academic literacies. Many of the evaluated studies in this review are examples of initiatives from individual higher education institutions, and subsequently, levels of evidence and generalizability are challenging to establish. We encourage integration of teaching and learning activities targeting parallel learning of course materials and development of academic literacies beyond study skills. Furthermore, teamwork and interprofessional collaboration are crucial in designing and developing teaching and learning activities corresponding to multiple dimensions of learning. Embracing epistemic complexity and diversity and choosing strategic work with academic literacies may provide a starting point toward realizing sustainable development goals and widening participation.

## Supplementary Information

**Additional file 1.**

**Additional file 2.**

## Data Availability

The datasets used and/or analyzed during the current study are available from the corresponding author on reasonable request.

## References

[1] Whitehead D (2002). The academic writing experiences of a group of student nurses: a phenomenological study. J Adv Nurs.

[2] Nambiar RMK. Enhancing academic literacy among tertiary learners: A Malaysian experience. J Tea Ling Lit. 2007;13:77–94.

[3] Lea MR, Street BV (2006). The “academic literacies” model: Theory and applications. Theor Pract.

[4] Jefferies D, McNally S, Roberts K, Wallace A, Stunden A, D’Souza S (2018). The importance of academic literacy for undergraduate nursing students and its relationship to future professional clinical practice: A systematic review. Nurs Educ Today.

[5] United Nations. Transforming Our World: The 2030 Agenda for Sustainable Development. In: Affairs DoEaS, editor. New York 2015. p. 41.

[6] Boughey C, McKenna S (2017). Academic literacy and the decontextualized learner. Crit Stud Teach Learn.

[7] Borglin G (2012). Promoting critical thinking and academic writing skills in nurse education. Nurs Educ Today.

[8] Lum L, Alqazli M, Englander K (2018). Academic literacy requirements of health professions programs: Challenges for ESL students. Tesl Can J.

[9] Christenbury L, Bomer R, Smagorinsky P (2009). Handbook of adolescents’ literacy.

[10] Lea MR, Street BV (1998). Student writing in higher education: An academic literacies approach. Stud High Edu.

[11] Marginson S (2016). The wordlwide trend to high participation higher education: dynamics of social stratification in inclusive systems. HighEdu.

[12] Calderon A (2018). Massification of higher education revisited.

[13] Holschuh JP (2019). College reading and studying: The complexity of academic literacy task demands. J Adolesc Adult Lit.

[14] Bornschlegl M, Cashman D (2019). Considering the role of the distance student experience in student satisfaction and retention. Open Learn.

[15] Sage AJ, Cervato C, Genschel U, Ogilvie CA (2021). Combining academics and social engagement: A major-specific early alert method to counter student attrition in science, technology, engineering, and mathematics. J Coll Stud Retent-R.

[16] Woods CS, Park T, Hu SP, Jones TB (2019). Reading, writing, and English course pathways when developmental education is optional: Course enrollment and success for underprepared first-time-in-college students. Community Coll J Res.

[17] Cagliero L, Canale L, Farinetti L, Baralis E, Venuto E (2021). Predicting student academic performance by means of associative classification. Appl Sci-Basel.

[18] Crowther P, Briant S (2021). Predicting academic success: A longitudinal study of university design students. Int J Art Des Educ.

[19] Arksey H, O’Malley L (2005). Scoping studies: towards a methodological framework. International Jnl Soc Res Metho.

[20] Tricco AC, Lillie E, Zarin W, O’Brien KK, Colquhoun H, Levac D (2018). PRISMA extension for scoping reviews (PRISMA-ScR): Checklist and explanation. Ann Intern Med.

[21] Ouzzani M, Hammady H, Fedorowicz Z, Elmagarmid A (2016). Rayyan-a web and mobile app for systematic reviews. Syst Rev.

[22] Braun V, Clarke V (2006). Using thematic analysis in psychology. Qual Res Psych.

[23] Cheng CY, Liou SR, Hsu TH, Pan MY, Liu HC, Chang CH (2014). Preparing nursing students to be competent for future professional practice: Applying the team-based learning teaching strategy. Jnl Prof Nurs.

[24] Daniels AD, Jooste K (2018). Support of students by academics in a nursing foundation programme at a university in the Western Cape. Curationis.

[25] Gordon-Handler L, Dimitropoulou K, Hassan L, Masaracchio M, Waldman-Levi A (2019). exploration of graduate health care student writing skills using a transformational learning approach to a literacy enrichment program. J Allied Health.

[26] Havery C, Townsend L, Johnson A, Doab A (2019). Professional development for teachers of nursing students for whom English is an additional language: A reflection on practices. Nurs Educ Pract.

[27] Hillege SP, Catterall J, Beale BL, Stewart L (2014). Discipline matters: Embedding academic literacies into an undergraduate nursing program. Nurs Educ Pract.

[28] McGowan BS (2019). Reimagining information literacy instruction in an evidence-based practice nursing course for undergraduate students. J Med Libr Assoc.

[29] Palmer L, Levett-Jones T, Smith R (2018). First year students’ perceptions of academic literacies preparedness and embedded diagnostic assessment. Stud Success.

[30] Rose D, Rose M, Farrington S, Page S (2008). Scaffolding academic literacy with indigenous health sciences students: An evaluative study. J Engl Acad Purp.

[31] Tarrant M, Dodgson JE, Law BVKK (2008). A curricular approach to improve the information literacy and academic writing skills of part-time post-registration nursing students in Hong Kong. Nurs Educ Today.

[32] Wette R (2019). Embedded provision to develop source-based writing skills in a Year 1 health sciences course: How can the academic literacy developer contribute?. Engl Specif Purp.

[33] Zanin-Yost A, Dillen C (2019). Connecting past to future needs: Nursing faculty and librarian collaboration to support students’ academic success. J Libr Adm.

[34] Faisal R, Khalil ur R, Bahadur S, Shinwari L (2016). Problem-based learning in comparison with lecture-based learning among medical students. J Pak Med Assoc.

[35] Busebaia TJA, John B. Can flipped classroom enhance class engagement and academic performance among undergraduate pediatric nursing students? A mixed-methods study. Res Pract Tech Enhan Learn. 2020;15(4):1–16.

[36] Asknes E (2017). Using quantitative literacy to enhance critical thinking skills in undergraduate nursing students. Jnl Nurs Educ.

[37] McMillan LR, Raines K (2011). Using the “Write” resources: nursing student evaluation of an interdisciplinary collaboration using a pofessional writing assignment. Jnl Nurs Educ.

[38] Abdolhosseini A, Keikhavani S, Hasel KM (2011). The effect of instructing cognitive and metacognitive strategies on the academic progress of Ilam Medical University students. Psych Res.

[39] Cronin C, Hawthorne C (2019). ’Poetry in motion’ a place in the classroom: Using poetry to develop writing confidence and reflective skills. Nurs Educ Today.

[40] Heatley S, Allibone L, Ooms A, Burke L, Akroyd K (2011). Providing writing and language support for students who have English as second language - a pilot study. J Voc Educ Tr.

[41] Igbo IN, Straker KC, Landson MJ, Symes L, Bernard LF, Hughes LA (2011). An innovative, multidisciplinary strategy to improve retention of nursing students from disadvantaged backgrounds. Nurs Educ Perspect.

[42] Sahoo S, Mohammed CA (2018). Fostering critical thinking and collaborative learning skills among medical students through a research protocol writing activity in the curriculum. Korean J Med Educ.

[43] Bailey P, Derbyshire J, Harding A, Middleton A, Rayson K, Syson L (2007). Assessing the impact of a study skills programme on the academic development of nursing diploma students at Northumbria University, UK. Health Info Libr J.

[44] Crawford T, Candlin S (2013). Investigating the language needs of culturally and linguistically diverse nursing students to assist their completion of the bachelor of nursing programme to become safe and effective practitioners. Nurs Educ Today.

[45] Grzyb K, Snyder W, Field KG. Learning to write like a scientist: A writing-intensive course for microbiology/health science students. J Microbiol Biol Educ. 2018;19(1):1–8. 10.1128/jmbe.v19i1.1338 PMC5969401 29904515

[46] Yu WW, Cheng CY, Lin CC, Wang J (2013). Fostering nursing students’ informatics competencies via a Web-based information literacy course. J Curr Teach.

[47] Griffiths L, Nicolls B (2010). e-Support4U: An evaluation of academic writing skills support in practice. Nurse Educ Pract.

[48] Hammond K (2018). "I need it now!“ Developing a formulaic frame phrasebank for a specific writing assessment: Student perceptions and recommendations. J Engl Acad Purp.

[49] Harrison S, LeBlanc N (2016). Method simple: An electronic interactive tool helping nursing students prepare for written and oral presentation. Nurs Educ Today.

[50] Latham CL, Ahern N (2013). Professional writing in nursing education: creating an academic-community writing center. J Nurs Educ.

[51] Miller LC, Russell CL, Cheng AL, Zembles S (2018). Testing the efficacy of a scaffolded writing intervention with online degree-completion nursing students: A quasi-experimental design. Nurs Educ Pract.

[52] Ooms A, Fergy S, Marks-Maran D, Burke L, Sheehy K (2013). Providing learning support to nursing students: A study of two universities. Nurs Educ Pract.

[53] Wolf DM, Linh P (2019). Studying in the United States: Language learning challenges, strategies, and support services. J Int Students.

[54] Lillis T, Scott M (2007). Defining academic literacies research: issues of epistemology, ideology and strategy. J Appl Ling.

[55] Shah M (2016). Widening higher education participation A global perspective.

[56] Heaslip V, Board M, Duckworth V, Thomas L (2017). Widening participation in nurse education: An integrative literature review. Nurs Educ Today.

[57] Barnett R (2012). Learning for an unknown future. High Educ Res Dev.

[58] Balloo K, Pauli R, Worrell M (2017). Undergraduates’ personal circumstances, expectations and reasons for attending university. Stud High Educ.

[59] Schlechter M, Milevsky A (2010). Parental level of education: associations with psychological well-being, academic achievement and reasons for pursuing higher education in adolescence. Educ Psychol-Uk.

[60] Rodriguez-Hernandez CF, Cascallar E, Kyndt E (2020). Socio-economic status and academic performance in higher education: A systematic review. Educ Res Rev.

[61] Sianou-Kyrgiou E, Tsiplakides I (2011). Similar performance, but different choices: social class and higher education choice in Greece. Stud High Educ.

[62] Lozano JF, Boni A, Peris J, Hueso A (2012). Competencies in higher education: A critical analysis from the capabilities approach. J Philos Educ.

[63] Okasha S (2002). Philosophy of Science. A very short introduction.

[64] Russell B (2001). The problems of philosophy.

[65] Seats MR. The voice(s) of reason: conceptual challenges for the decolonization of knowledge in global higher education. Teach High Educ. 2020. p. 1–17.

[66] Ryder AJ, Reason RD, Mitchell JJ, Gillon K, Hemer KM (2016). Climate for learning and students’ openness to diversity and challenge: A critical role for faculty. J Divers High Educ.

[67] Hall B. Knowledge democracy, higher education and engagement: Renegociating the social contract. In: Kariwo M, Guonko T, Nungu M, editors. A comparative analysis of higher education systems: Issues, challenges and dilemmas: Brill; 2014. p. 141–52. https://www.tandfonline.com/doi/full/10.1080/13562517.2020.1729725.

[68] Lockwood C, dos Santos KB, Pap R (2019). Practical guidance for knowledge synthesis: Scoping review methods. Asian Nurs Res.

