# Supplementary material for: Methods and strategies to promote academic literacies in health professions: a scoping review
Source: BMC Med Educ. 2022 Jun 1;22:418. doi: 10.1186/s12909-022-03288-9 (PMC9156819; doi:10.1186/s12909-022-03288-9)
Supplement: Supplementary file 2 — Additional file 2. [file 12909_2022_3288_MOESM2_ESM.docx]

Additional file 2. Matrix of included studies; author, aim, method, interventions and findings

|  | **Author, year, country** | **Aim** | **Method** | **Intervention** | **Main findings** |
| --- | --- | --- | --- | --- | --- |
| 1 | Abdolhosseini, A., Keikhvani, S., & Hasel, K. M.  (2011)   Iran | To study the effect of instructing cognitive and metacognitive strategies on the academic progress. | **Design:** Experimental  **Sample:** Students (n=120) from four majors (nursing, occupational health, family health, medicine) at Ilam University, Iran were selected randomly.  **Data collection:** Pre-test and post-test of academic progress (general point average [GPA]).  **Analysis:** Descriptive and inferential statistics. | **Intervention:** Instruction of cognitive and metacognitive strategies including study skills in six sessions; strategies comprised  - repetition and revision  - semantic extension  - organizing  - planning  - control & supervision  - ordering. | The results showed that the students improved the GPA score significantly (p=0.000) post-test compared to pre-test. The female students improved significantly (p=0.02) more than the male students. The GPA differed significantly (p=0.011) between the majors, but not stated in what way. |
| 2 | Asknes, E.  (2017)  USA | To evaluate a teaching strategy designed to enhance critical thinking skills in undergraduate nursing students. | **Design:** Experimental  **Sample:** Students (n=23) in a 7 weeks maternal-newborn course.  **Data collection:** Formative and summative analysis of student progress during and after. Interviews.  **Analysis:** Descriptive; Compilation of examination results. | **Intervention:** Three data-based contextual assignments  requiring critical thinking. | Each assignment revealed enhanced and ongoing development of critical-thinking and problem-solving skills. Three students with self-reported “math anxiety” expressed feeling more confident when working with numbers. ­ |
| 3 | Bailey, P., Derbyshire, J., Harding, A., Middleton, A., Rayson, K., & Syson; L.  (2007)  United Kingdom | To evaluate the impact of workshops supplementing online instruction on study skills on students’ study and writing skills, and level of information literacy. | **Design:** Quasi-experimental  Qualitative and quantitative  **Sample:** Pre-registration nursing students (n=28) needing support with information literacy and study skills.  **Data collection:** Pre-and post focus groups; questionnaire, grades and diagnostic test.  **Analysis:** Not specified. | **Intervention:** Four series of workshops were offered, using Skills Plus. Issues for the workshops was raised by the students, for example essay writing and literature searching. | Students who attended at least one workshop improved their academic grade in their next assignment. Confidence level and information literacy, including referencing skills, improved. Students identified improvement in ability to find quality information, to understand assignments, and to proofread before submission. Face to face interaction in the workshops to answer questions was valued. |
| 4 | Busebaia, T. J. A. & John, B.  (2020)  Bahrein | To assess the nursing students’ class engagement and academic performance using flipped classroom. | **Design:** Action research using mixed methods for triangulation.  **Sample:** Students (n=26) in a fourth-year Bachelor of Science in Nursing nursing program.  **Data collection:** checklists to observe and assess students’ class engagement; quizzes for measuring class performance; focus group interviews (5 students).  **Analysis:** Descriptive and inferential statistics, content analysis. | **Intervention:** Flipped classroom instead of traditional lecturing; academic performance interpreted as indicative of academic literacies. | Grades significantly (p=0.001) improved after flipped classroom as well as higher classroom engagement. Students expressed learning and understanding better. Preparation facilitated using class time better, interactive aspects superior to standard lecturing. Comprehensive learning promoted, aligned with future practice. |
| 5 | Cheng, C.-Y., Liou, S.-R., Hsu, T.-H., Pan, M.-Y., Liu, H.-C., & Chang, C.-H.  (2014)  Taiwan | To evaluate the effect of Team-based Learning (TBL) on students' learning outcomes and behaviors | **Design:** Experimental  **Sample:** Nursing students (n=387 out of 399 contacted) in two- and four year programs at a university in Taiwan.  **Data collection:** Questionnaires, pre-post test  **Analysis:** Descriptive and inferential statistics | **Intervention:** Course design was built on Team Based Learning with interaction among students for studying and learning. | Team Based Learning improved the learning behaviors of students, including class engagement and self-directed learning  (p<0.001). The average final examination score  was significantly higher than individual readiness assurance test scores  (p<0.001), indicating that TBL improved individual  academic performance through collaborative learning. |
| 6 | Crawford, T. & Candlin, S.  (2013)  Australia | To identify the language needs of culturally and linguistically diverse students and evaluate the English language support program to develop appropriate strategies and assist academic progression and clinical communication skills. | **Design:** Action research  **Sample:** Bachelor of Science in Nursing second- and third-year nursing students (n=8) with culturally and linguistically diverse background; convenience sample out of 45 possible students.  **Data collection:** Semi-structured individual interviews  **Analysis:** Content analysis | **Intervention:** English lanuage support program for students with English as second language. Content not specified. | Academic writing stressful and continuous support is needed. Language immersion important. Nursing content was relevant for language studies. Time constraints was a challenge for attendance. Varying lecture style was crucial. |
| 7 | Cronin, C., & Hawthorne, C.  (2019)  United Kingdom | To explore how classroom- based poetry writing activities might support students in developing their skills as student-writers and reflective practitioners. | **Design:** Experimental, multiple case study  **Sample:** Pre-nursing students (n=25) in health science foundation course; selection not clear, possibly all students in the class, i.e. mandatory.  **Data collection:** Observations and retrospective analysis of a poetry writing activity.  **Analysis:** Thematic analysis | **Intervention:** This writing session was scheduled in the first few weeks of the program with the aim of developing reflective skills and building students' confidence about their writing abilities. One poetry writing session after listening to a poem and looking at a photograph. Students had 45 min to write a poem about nursing practice. | Poetry writing tool to put words on complex phenomena. Most students were nervous before the session, happy after. Poetry allowed expressing feelings and emotions; some were worried that poems reveal too much compared to formal assignments. Poetry can be used to capture complex phenomena and be a tool for reflective practice. Early introduction in education may promote reflective practice. |
| 8 | Daniels, A. D. & Jooste, K.  (2018)  South Africa | To explore and describe experiences of students about the support from lecturers in the nursing foundation program. | **Design:** Qualitative  **Sample:** Students (n=8) in foundation program; purposive sampling; ages 20-51; first languages: isiXhosa (n=3), seTswana (n=2), English (n=2), Afrikaans (n=1).  **Data collection:** Qualitative interviews and observation  **Analysis:** Thematic analysis | **Intervention:** The university created a five-year foundation nursing program to meet needs in disadvantaged students of color; to counter post-apartheid racial and class inequalities; the first year is completed in two years, then on to regular program. | Students found that an overall supportive learning environment was crucial; experienced lecturer support contributed to academic success; trusting relationships and respect were key aspects; caring for students, advising and being both persistent and patient. Second theme was peer support that extended outside program and a sense of comradery in the peer group. Lecturer support resulted in academic growth. |
| 9 | Faisal, R., Rehman, K., Bahadur, S., & Shinwari, L. (2016)  Pakistan | To compare performance of medical students exposed to problem-based learning and lecture-based learning. | **Design:** Experimental, pre-post test  **Sample:** Medical students (n=146) in the third year, randomized into two groups (n=73; n=73); selection not clear, possibly all students in the classes  **Data collection:** Test results of course.  **Analysis:** Descriptive and inferential statistics | **Intervention:** Same course given in two different ways; one traditional lecture one problem-based; evaluated by same test of course results. | Problem-based learning was more effective than lecture-based learning in the academic performance of medical students; mean score PBL 3.2 (sd 0.8) and lecture based 2.7 (sd 0.8), (p= 0.0001). |
| 10 | Gordon-Handler. L., Dimitropoulou, K., Hassan, L., Masaracchio,. M., & Waldman-Levi , A.  (2019)  USA | The aim was to examine the impact and satisfaction of a transformational learning approach in a literacy-enriched health professions program on student’s writing skills. | **Design:** Non-experimental, cross-sectional, correlational  **Sample:** Three mixed and three mono-cultural cohorts of occupational therapy students (n=165) from culturally diverse ethnic, socioeconomic and educational backgrounds  **Data collection:** Pre- and-post questionnaire ACT-ASSE, (mathematics, English, reading), writing test score and student satisfaction survey (post) across three different years  **Analysis**: Descriptive and inferential statistics | **Intervention:** Six consecutive one-hour sessions took place within the first half of each class session. The literacy-enriched program was based on transfor­mative learning theories and was delivered by program faculty and writing tutors from the university writing center. | Significant increase in the pre- and-post ASSET scores and writing scores, except for two cohorts. Aggregated student satisfaction in both cohorts over the 3-year period showed that many students reported sat­isfaction with the writing program regarding ability to revise, edit, proofread, and paraphrase content. |
| 11 | Griffith, L., & Nicolls, B.  (2010)  United Kingdom | The main aim was to encourage deeper learning through more active/interactive approaches between tutor-student and student-student, and creating a platform for mutual support | **Design:** Experimental  **Sample:** Nursing students (n=17) entering pre-registration through the widening participation route; convenience sampling.  **Data collection:**  The online questionnaire and the e-Support4U program evaluation Blog; participants’ perceptions of value and effectiveness  **Analysis**: Qualitative, descriptive and narrative | **Intervention:** e-Support 4U by electronic technology with a blended learning approach, combining constructivist theory, e-communication tools, like e-mail, Blogs, Wikis and the discussion board. Students received 20 guided learning hours including an introduction delivered via Blackboard and 10 hours of independent study. | Evaluation suggests that the approach to academic writing may have contributed to a 100% pass rate. Findings showed a 100% positive response to the online academic support to the students whilst on their practice placements. The Wiki pages were rated highly by three students. All participants felt that continuous development of academic writing skills was possible in the support tool. |
| 12 | Grzyb, K., Snyders,.W,. & Field, K.  (2018)  USA | The objective was to assess the overall success of a writing intense course and the effectiveness of each course element. | **Design:** Experimental, pre-post test  **Sample:** Bioscience students (n=60)  **Data collection:** Pre- and post-survey of students’ experiences; training and instruction; and skills and knowledge.  **Analysis**: Descriptive statistics | **Intervention:** Development of a writing intensive course to teach upper-division students how to improve science-specific writing skills within their specific disciplines; Course promotes both writing to learn and gaining expertise in discipline-specific writing genres. | The students’ confidence levels rose in both skills and knowledge. Discipline - specific knowledge had been acquired through writing activities. Level of interest in learning the conventions of the genre and in seeking out detailed information about emerging infectious diseases increased. |
| 13 | Hammond, K.  (2017)  New Zeeland | The study aimed to redesign, implement, evaluate, and develop the targeted phrase bank through iterative cycles.  Research questions:  1.How do the students perceive the usefulness of the phrase bank?  2.What are students’ suggestions for improvement? | **Design:** Action research, mixed methods  **Sample:** First-year health science students in two cycles (n=285, 40%; n=213, 27%); interviews (n=16; n=5) strategic selection of participants; 80% English first language speakers.  **Data collection:** Questionnaires and interviews  **Analysis:** Descriptive statistics, content analysis and thematic analysis | **Intervention:** Providing support for academic writing through an assignment-specific phrase bank; phrase bank developed from the course textbook and students’ papers on human development, and teacher input; student feedback on the phrase bank and recommendations for improvement; second cycle, further development of phrase bank based on students’ recommendations. | Nearly one-fifth of the students (18.8%) used the Cycle 1 phrase bank; twelve of the 16 interviewed students preferred a targeted phrase bank over a general resource, more relevant to assessment, type of writing required and timesaving; students reported the phrase bank in Cycle 2 was helpful for writing and thinking, more positive evaluation and uptake from students; many students found the Cycle 2 phrase bank useful (49.3%); some reported difficulties in understanding (25.1%); phrase bank “too wordy”. |
| 14 | Harrison, S,. & LeBlanc, N.  (2016)  Canada | To discover how students learn to use the tool, what barriers affect proper use of the tool, and what role faculty plays in ensuring students' successful use of the tool. The study also sought to explore students' perception in regard to the contribution of the tool to improve their academic work. | **Design:** Experimental.  **Sample:** Nursing students (n=14) and faculty (n=6) who adopt the web-based tool (Method SIMPLE) in their courses.  **Data collection:** Three focus groups interviews; Likert scale survey; three open-ended questions regarding aspects, most liked and least useful.  **Analysis:** Thematic, descriptive analysis using NVivo 8; deductive elements based on the structure of the Method SIMPLE tool. | **Intervention:** Introduction of a web based interactive tool (Method SIMPLE) with access at any time; tool consist of six modules: Sorts of information, Information being communicated, My audience, Path required, Language required, Excellence; students exposed to tool in their first year; faculty instructs students to refer to the tool in difficulties preparing oral and written presentations. | Students described being a little overwhelmed; user-friendly tool with various examples, especially referencing; more reference examples beneficial; faculty appreciated tool for ease of use and accessibility. |
| 15 | Havery, C,. Townsend, L,. Johnson, A,. & Doab, A.  (2019)  Australia | To enable English as additional language students better transition to Australian university studies by integrating an explicit focus on language development within the subject content. | **Design** Experimental, descriptive  **Sample:** Teachers, nursing academic and one language educator (n=5)  **Data collection:** Reflective journals discussed in weekly meetings; emails  **Analysis** Interpretive description | **Intervention:** Pilot project where a language academic and a group of nursing academics, adopted a clinical supervision model to problematize subject content and pedagogic practices; professional development program including weekly meetings and written reflective journals. | Main challenges faced by academic teachers were covering the content of the subject, responding to students' learning styles, and tutors’ lack of confidence in teaching classes of English as additional language students. |
| 16 | Heatley, S,. Allibone, S,. Ooms, A,. Burke, L,. & Akroyd, K.  (2011)  UK | Evaluate the effectiveness of the writing support offered from the perspective of the students and evaluate the impact on lecturers of attending the staff workshops on the approach to teaching students who have English as a second language. | **Design:** Descriptive case study  **Sample:** Three groups of nursing students (n=37)  **Data collection:** Questionnaire with mix of quantitative (Likert scale) and qualitative questions at end of each session  **Analysis:** Qualitative, descriptive; according to framework | **Intervention:** Writing support to students who have English as a second language (ESL); learning approach by facilitators based on constructivism with booster workshop sessions in study/academic writing skills; staff workshops focusing on identification of writing errors and constructive written feedback to students. | Three themes emerged from the post-registration and pre-registration student data: reasons for using the support service, appreciated aspects, and suggestions for improvements; most students post registration found the support ‘very useful’ or ‘useful’, and 24/26 students felt improved confidence; students particularly valued individual consultations with the tutor. |
| 17 | Hillege, S.P., Caterall, J,. Neale, B.L,. & Stewart,L.  (2014)  Australia | The aim was to evaluate the embedded literacy strategy in a core subject in the Bachelor of Nursing program. | **Design:** Experimental, pre-post test  **Sample:** Enrolled nursing students (n=747) screened for and identified as needing support; invited (n=147), students who attended tutorials (n=107).  **Data collection:** A Post Enrollment Language Assessment (PELA) questionnaire before tutorial; survey evaluating the effectiveness of the developed resources after tutorial  **Analysis:** Descriptive statistics | **Intervention:** Teaching team made changes to existing course by embedding literacy; more active student opportunities, more dialogue related to academic literacies, more writing and feedback; all students screened and identified students encouraged to participate in literacy tutorials for specific subjects. | Students with lower level language skills who attended the streamed tutorial with additional literacy support showed a greater improvement in their written communication than those with similar language proficiency who attended non-streamed tutorials. |
| 18 | Igbo,K., Starker, C., Landson, M.J., Symes, L., Bernard, L.F., Hughes, L.A., & Caroll, T.L. (2011)  USA | Describe how the interdisciplinary, collaborative efforts in the nine-month retention program contributed to the retention and success of nursing students | **Design:** Experimental  **Sample:** Identified by federal criteria for disadvantaged nursing students and invited at admission; participants (n= 27, 39, 39) over three year period;  **Data collection:** Student comments; progression through education  **Analysis:** Descriptive evaluation | **Intervention:** A retention program (for 9 months), with a multidisciplinary team in three schools; various activities in workshop format; study skills (preparing for lectures, taking notes, critical thinking, test-taking strategies), professional socialization and career coaching; activities two hours per week throughout the first academic year of nursing school. | Overall average completion rate increased; participating students reported increase in grade-point-average and confidence; comradery of program appreciated; multidisciplinary team helped 76 percent of high-risk students persist in the nursing program; proactive role to address student needs is recommended, also adding social component to future initiatives. |
| 19 | Latham, C.L., & Ahern, N.  (2013)  USA | Describe a comprehensive approach to assessing and supporting nursing students’ writing skills; Evaluation of an Academic Community Writing Center | **Design**: Action research  **Sample:** Nursing students (n=83) representing a variety of ethnicities; all students invited, took test (n=180); minority or underrepresented students targeted  **Data collection:** Focus groups (n=10) 10-15 students in each focus group; student feedback to evaluate tutoring partnerships  **Analysis:** Descriptive evaluation | **Intervention:** Two prelicensure programs initiated; comprehensive assessment of writing quality by artificial intelligence writing tool, ACCUPLACER®; student support needs; three strategies to address writing skill deficiencies: changing the order of courses, incorporating more writing instruction in curriculum, and implementing a writing center to improve student writing. | Students reported positive experiences with the writing tutors; writing in 68% remained the same or improved; APA referencing especially challenging; collaboration with writing center positive in terms of anecdotal comments; student evaluations shared with tutors each Spring. |
| 20 | McGowan, B.S.  (2019)  USA | To describe the redesign process for an undergraduate evidence-based practice (EBP) nursing course in which the librarian serves as both co-instructor and co-instructional designer. | **Design:** Case report, pre- and post test  **Sample:** Nursing students pre-course (n=25), post-course (n=54)  **Data collection:** Pre- and post course surveys  **Analysis:** Descriptive statistics | **Intervention:** Redesign of the undergraduate evidence-based practice (EBP) nursing course; Writing assignment based on health-related topic, librarian both as co-instructor and co-instructional designer; course included: research process; strengths and limitations of research articles in relation to EBP; build student confidence abilities for information literacy, data management, and scholarly communication competencies. | The overwhelming preferred learning medium was in-class lectures and activities. Improved student engagement throughout the course. Significantly increased student confidence, reflected by lower ratings, in using APA style formatting after the course (t(41)=3.02, p=0.004). All other confidence ratings, however, were unchanged between pre- and post-course surveys |
| 21 | McMillan, L. R., Raines, K.  (2011)  USA | The purpose was to describe resource support and evaluation strategies used in a professional paper writing assignment in a baccalaureate nursing program. | **Design:** Case study  **Sample:** Nursing students (n=46)  **Data collection:** A survey (Writing Assignment Resource Evaluation) was developed at the end of the semester  **Data analysis:** Descriptive statistics | **Intervention:** Development of a writing assignment designed to enhance academic writing skills. Focus group meetings with four librarians to develop the 1-hour session. Session with five students and one librarian devoted to information literacy sources. Focus of creating thesis statement and searching for relevant sources. Librarians involved in final grading regarding quality of sources. Peer review component where students provided feedback to each other. | Students found collaboration with librarians helpful in essay writing. Feedback on draft from tutor contributed to learning (63%). Peer review and collaborative learning positive and contributed to learning (85%). Students found classroom instruction constructive (98%). Faculty found paper quality improved. |
| 22 | Miller, L.C., Russell, L.C., , An-Lin Cheng, A-L., Zembles, S.  (2018).  USA | The purpose of this pre-post quasi-experimental study was to compare implementation of a scaffolded sequence of writing assignments (intervention) to typical writing assignments (comparison) in final coursework for baccalaureate nursing (BSN) completion students. | **Design:** Experimental with control, pre-post test  **Sample:** Convenience sample of 78 of 120 (65%) BSN-completion student; intervention group 51/59 students (86%); control group 27/61 (44%)  **Data collection:** Assessment of writing self-efficacy  **Analysis:** Descriptive and inferential statistics; using IBM SPSS software | **Intervention:** The intervention was writing intensive, using a scaffolded sequence of assignments requiring students to work with various genres of writing. Students created persuasive written arguments related to a selected evidence-based practice question, moving from simple writing tasks to more complex writing skills over the semester. | Implementation of a planned sequence of writing could give more competent writers. All students in the two groups demonstrated improved writing self-efficacy on 18 of 19 items (p = < 0.001). Writing competency, average change scores from pre-to post-writing were significant for all students on both the Holistic scale and the summed Trait scale (both p's = < 0.001. Two of the seven traits organization and voice significantly improved for the intervention group compared to the comparison group. (p = 0.003) and (p = 0.007). |
| 23 | Ooms, A., Fergy, S., Di Marks-Maran, Di., Burke, L., Sheehy, K.  (2013).  United Kingdom | The aim was to measure the perceptions of students of the use and usefulness of the support mechanisms provided by their university. | **Design:** Mixed methods evaluation  **Sample:** Nursing students (n=812); University A (n=433), University B (n=379)  **Data collection:** Questionnaires with Likert-style and open-ended questions.  **Dataanalysis:** Descriptive statistics using SPSS v. 18; qualitative, descriptive, deductive | **Intervention:** Evaluative study of the support services provided to undergraduate nursing students in two universities in the United Kingdom. Data were collected by a mapping exercise at both universities to identify and describe in depth all forms of existing student support across each university (academic, pastoral, study skills and key skills support). | Results showed that support services which has the most impact on student success are program leaders, module teachers, smaller study groups and support sessions. Students with English as second language and non-traditional entry qualifications, numeracy and academic literacy was very valuable. Students found that support helped increase confidence, reduce anxiety and was helpful. Communication issues with support services was identified as needing improvement. |
| 24 | Palmer, L, Levett-Jones, T, Smith, R.  (2018)  Australia | Exploring students’ perceptions of the combined strategy of embedding academic literacies education and diagnostic assessment using the MASUS, and whether they thought this was effective in developing their academic literacies capability during their first year. | **Design:** Mixed methods survey  **Sample:** First year undergraduate nursing students 165 of 458 completed the survey (response rate=36%)  **Data collection:** 20-item survey; demographic data, closed and open-ended questions  **Data analysis:** Descriptive statistics using Microsoft Excel; qualitative, descriptive content analysis | **Intervention:** Embedding academic literacy education and diagnostic assessment in course curricula. Focus in diagnostics was use of source material, structure and development of answer, writing style and grammatical correctness. | The findings showed that embedded academic literacies in combination with the MASUS diagnostic assessment were beneficial to students. Constructive feedback and insights into expectations and requirements was appreciated. |
| 25 | Rose, D., Rose, M., Farrington, S. & Page, S.  (2008)  Australia | The purpose of the research was to test the efficacy of the Scaffolding Academic Literacy pedagogy with Indigenous students in the context of such a health sciences curriculum. | **Design:** Action research evaluation  **Sample:** Health science students (n=25); one-year Preparatory program (n=5), first year of Bachelor's degree course (n=8), second year of the Bachelor degree course (n=12)  **Data collection:** Pre-test to evaluate students’ academic writing skills; post-test analysis of all writing samples  **Data analysis:** Comparative analysis combining qualitative analysis with a numerical score | **Intervention:** Incorporating teaching of literacy skills into teaching in the academic curriculum. Pre-test to evaluate students’ academic writing skills. Implementing new course structure. Written assessment at conclusion of semesters one  and two. | Students’ academic literacy improved through supporting the students to read and write academic texts as stated in the curriculum. Integrating reading and writing skills into the academic curriculum accelerated academic literacy development including a better understanding of their field of study. |
| 26 | Sahoo, S & Mohammed, C. A.  (2018)  Malaysia | To analyze the effect of academic writing and journal critiquing as educational approaches in improving critical thinking and collaborative learning among undergraduate medical students. | **Design:** A mixed methods study was designed to explore the possible role of collaborative research proposal writing in enhancing critical thinking and collaborative learning.  **Sample:** Fourth year medical students (n=188) during their clinical posting in ophthalmology.  **Data collection:** Research protocols developed by students in small groups and written reflective summaries in academic portfolios about the activity.  **Data analysis:** Qualitative analyses student reflections; descriptive statistics | **Intervention:** Students worked in small groups and developed research protocols through an evidence- based approach. This was followed by writing reflective summaries in academic portfolios about the activity undertaken. | Students improved critical thinking skills and collaborative learning after the intervention. The model helped all students to apply concepts into new situations in form of designing their own study, which reflected in their cognitive skills. Students all agreed that course was useful in terms of collaborative learning and writing of research proposal. |
| 27 | Tarrant, M., Dodgson, J.E., Beatrice Law, B.  (2008)  Hong Kong, China | The aim of this curricular intervention was to develop and strengthen post-registration nursing students’ information literacy skills. | **Design:** Evaluation design, pre- post test  **Sample:** Three successive cohorts of nursing students (n=159/194 possible); 85% female; mean age 34  **Data collection:** Pre- and post-test self-administered questionnaires regarding information literacy and academic writing  **Data analysis:** Descriptive and inferential statistics | **Intervention:** An introductory module on developing students’ skills in database searching, locating and retrieving the literature, critical appraisal of nursing and health studies, and scholarly writing. Nursing faculty collaboration with librarians. | Structured, flexible, integrated learning activities in core curriculum improved the academic information literacy significantly. Confidence in ability to locate, retrieve and analyze health-related data improved. All skills showed statistically significant improvements in perceived competency on both the post-test and final test (p< .001) Overall knowledge scores increased in the final test (p< .001). |
| 28 | Wette, R.  (2019)  New Zealand | To elicit the perspectives of lecturers, tutors, students, an academic literacy developer, and a library professional on an embedded provision to develop source-based writing in a health sciences course | **Design:** Mixed methods evaluation  **Sample:** Students in a health sciences course (n=280), lecturers (n=3), tutors (n=4), librarian (n=1)  **Data collection:** Student questionnaire (n=66, 24% completion rate); faculty interviews (n=13); observations, documents  **Data analysis:** Qualitative, inductive analysis; Descriptive statistics | **Intervention:** Students were provided support over the first semester in the form of documents, tutorials, online discussion and structured assignment tasks, as well as through embedded instruction offered collaboratively by subject lecturers, an academic literacy developer, and a library professional. | Discipline-specific instruction can connect academic literacy skills with disciplinary thinking and writing practices. Students’ evaluations showed that they found it motivating to be able to connect source-based writing learning with specific course objectives, content and assessment. Various forms were evaluated as relevant and helpful, there were several problems identified related to information-sharing and the need for explicit instruction in linguistic and communicative components aspects of academic writing. |
| 29 | Wolf, D.M. & Phung, L.  (2019)  United States | To explore the experiences of Chinese nurses when completing a graduate nursing degree taught in English (as a second language) in the United States over a 1-year period. | **Design:** Descriptive, exploratory case study, mixed methods  **Sample:** Bachelor’s-prepared Chinese nurses (n=8)  **Data collection:** Electronic survey, interview. Survey data were collected at three different points in time, and three interviews were conducted with each participant.  **Data analysis:** Qualitative descriptive using NVivo 11; Descriptive statistics using Microsoft Excel 2010 | No intervention; however, a strategy employed by university to promote academic writing (part of academic literacies) | The study identified participants’ difficulties with academic writing, mixed experiences with speaking, strategies for learning, and appreciation of the support services offered: tutoring workshops, small group learning and advice from professors was appreciated. |
| 30 | Yu, W.W., Cheng, C-Y., Lin, C.C., Wang, J.  (2013)  Taiwan | This study aimed to demonstrate nursing faculty’s efforts in the development of a web-based course to promote nursing students' information literacy competency. | **Design:** Experimental; testing and implementation of course  **Sample:** Nursing students (n=42; 53%) in their final year;  **Data collection:** 15-item self-report online survey; participants’ perceptions of web-mediated teaching strategy (8 questions); information literacy (7 questions).  **Analysis:** Descriptive statistics | **Intervention:** Development and implementation of web-based nursing information literacy course; 18-month course in years 2011-2012; phases: initial design, pilot-test and modification, final implementation; students evaluated through weekly journals, literature searching, website critique, discussion forum participation, and final project. | Students positive about computer-mediated learning and more knowledge and skills to apply in a caring context. Effective communication and knowledge building through web-based course. Helps lifelong learning and promotes self-learning. |
| 31 | Zanin-Yost, A. & Dillen, D.  (2019)  USA | To describe the collaborative process to integrate information literacy instruction into the Mental Health Nursing course and how the lessons learned were applied to the BSN program | **Design:** Case study evaluation  **Sample:** Undergraduate nursing students, first semester (n) not specified, second semester (n=27), third semester (n=38), fourth semester (n) not specified  **Data collection:** Semester three surveys (n=37); verbal feedback, faculty observations  **Data analysis:** Descriptive; not clearly outlined | **Intervention:** Evolution of a research assignment during four semesters in an undergraduate nursing course and development of the information literacy session; a variety of strategies, (workshops, checking drafts) from nursing faculty and librarians in collaboration to support students’ academic success. | The extended library session proved to be a good strategy for students to ask questions, initiate research and receive feedback. Close collaboration with the faculty allowed the liaison librarian to better understand faculty expectations and to gauge skills students needed to improve. Expectations of writing clearer to students and stress levels reduced. Faculty found student writing improved. Poster session appreciated by students learning from each other. |
